# A review of clinical pharmacogenetics Studies in African populations

**DOI:** 10.2217/pme-2019-0110

**Published:** 2020-03-03

**Authors:** Fouzia Radouani, Lyndon Zass, Yosr Hamdi, Jorge da Rocha, Reem Sallam, Sonia Abdelhak, Samah Ahmed, Maryame Azzouzi, Ichrak Benamri, Alia Benkahla, Balkiss Bouhaouala-Zahar, Melek Chaouch, Haifa Jmel, Rym Kefi, Ayoub Ksouri, Judit Kumuthini, Phumlani Masilela, Collen Masimirembwa, Houcemeddine Othman, Sumir Panji, Lilia Romdhane, Chaimae Samtal, Rania Sibira, Kais Ghedira, Faisal Fadlelmola, Samar Kamal Kassim, Nicola Mulder

**Affiliations:** 1 Research Department, Chlamydiae & Mycoplasmas Laboratory, Institut Pasteur du Maroc, Casablanca 20360, Morocco; 2 Computational Biology Division, Department of Integrative Biomedical Sciences, IDM, CIDRI Africa Wellcome trust Centre, University of Cape Town, South Africa; 3 Laboratory of Biomedical Genomics & Oncogenetics, Institut Pasteur de Tunis, Université Tunis El Manar, 13, Place Pasteur BP 74, 1002 Tunis, Belvédère, Tunisie; 4 Sydney Brenner Institute for Molecular Bioscience, University of The Witwatersrand, Johannesburg, South Africa; 5 Medical Biochemistry & Molecular Biology Department, Faculty of Medicine, Ain Shams University, Abbaseya, Cairo 11381, Egypt; 6 Centre for Bioinformatics & Systems Biology, Faculty of Science, University of Khartoum, 321 Khartoum, Sudan; 7 Faculty of Clinical & Industrial Pharmacy, National University, Khartoum, Sudan; 8 Systems & Data Engineering Team, National School of Applied Sciences of Tangier, Morocco; 9 Laboratory of Bioinformatics, Biomathematics & Biostatistics LR 16 IPT 09, Institute Pasteur de Tunis, Tunisia; 10 Laboratory of Venoms & Therapeutic Molecules, Pasteur Institute of Tunis, 13 Place Pasteur, BP74, Tunis Belvedere- University of Tunis El Manar, Tunisia; 11 H3ABioNet, Bioinformatics Department, Centre for Proteomic & Genomic Research, Cape Town, South Africa; 12 DMPK Department, African Institute of Biomedical Science & Technology, Harare, Zimbabwe; 13 Département des Sciences de la Vie, Faculté des Sciences de Bizerte, Université Carthage, 7021 Jarzouna, BP 21, Tunisie; 14 Biotechnology Laboratory, Faculty of Sciences Dhar El Mahraz, Sidi Mohammed Ben Abdellah University, Fez 30000, Morocco; 15 Department of Biology, University of Mohammed Premier, Oujda, Morocco; 16 Department of Biology Faculty of Sciences, University of Sidi Mohamed Ben Abdellah, Fez, Morocco; 17 Department of Neurosurgery, National Center For Neurological Sciences, Khartoum, Sudan

**Keywords:** Africa, communicable diseases, genetic variation, noncommunicable diseases, pharmacogenetics, pharmacogenomics, precision medicine

## Abstract

Effective interventions and treatments for complex diseases have been implemented globally, however, coverage in Africa has been comparatively lower due to lack of capacity, clinical applicability and knowledge on the genetic contribution to disease and treatment. Currently, there is a scarcity of genetic data on African populations, which have enormous genetic diversity. Pharmacogenomics studies have the potential to revolutionise treatment of diseases, therefore, African populations are likely to benefit from these approaches to identify likely responders, reduce adverse side effects and optimise drug dosing. This review discusses clinical pharmacogenetics studies conducted in African populations, focusing on studies that examined drug response in complex diseases relevant to healthcare. Several pharmacogenetics associations have emerged from African studies, as have gaps in knowledge.

There is huge genetic, cultural and lifestyle diversity among African populations that influence susceptibility to disease, disease progression and response to medical treatment employed against disease [1]. This applies to communicable and noncommunicable diseases, of which African countries face a huge burden of both. The challenges of the modern day patient compounded by the complexities associated with the poly-pharmacy requires careful management of dosages for the most optimal patient benefits while at the same time reducing the incidence of drug-induced adverse effects [1]. Infectious and parasitic diseases are highly prevalent and well-researched across Africa, however, there is growing interest in the increasing burden of noncommunicable diseases as well. Though effective interventions for these diseases have been implemented globally. Coverage in Africa has been comparatively low due to a lack of data, research and knowledge on African populations as well as funding, capacity and clinical applicability in this region [1,2]. Investigating the genetic influence of disease development, progression and treatment is further complicated in African populations, due to their great ethnolinguistic and genetic diversity [1,3].

The implementation of precision medicine in various medical fields has seen a global increase in recent times. Precision medicine describes a treatment approach which considers a patient’s genetics, behaviour, environment and lifestyle [4]. Pharmacogenomics, a branch of precision medicine, studies the influence of genomic variations on drug processing and response [5]. More specifically, pharmacogenetics looks specifically at the impact of variations in a single or few genes on drug response using genetic, epigenetic and nutrigenetic approaches [5]. For simplicity, we use these two terms interchangeably to refer to both single or multiple gene investigations. Studies in these fields have the potential to revolutionise the manner in which diseases are treated, emphasising the importance of studying these factors in African populations [3].

The use of pharmacogenetics as a tool for evidence-based medication management is gaining acceptance beyond academic research settings, with many users – individual patients, health professionals and medical plans – expressing interest in using pharmacogenetics tests to predict the efficacy and potential side effects of drug prescriptions. Unfortunately, pharmacogenetics and pharmacogenomics research in Africa is lagging behind international standards [5]. However, a few studies have been conducted and their outcomes are highlighted in this review. To implement pharmacogenetics into clinical practice in Africa, sharing of information and infrastructure support must be made available to researchers across the continent. Pharmacogenetics implementation requires digital storage and secure, prompt accessibility of information to authorised users, often with pharmacogenetics data embedded as part of an electronic health record system.

In this review, we investigated the scope of pharmacogenetics studies that have been conducted in African populations, and documented the relevant genotype–phenotype associations, focusing particularly on those that examined drug response in disease phenotypes relevant to African healthcare. The role of African genetic diversity and the opportunities for pharmacogenetics researchers are thus highlighted and will enable discovery of novel genetic mechanisms and validation of established markers. The review aimed to identify important genomic markers which can facilitate and guide precision medicine or precision public health in Africa. In addition, the review aimed to identify the current gaps in clinical pharmacogenetics and genomics research across the continent.

## Surveying the literature on pharmacogenetics studies

A systematic literature review was performed using several databases, including OvidMEDLINE, PubMed, Cochrane Library via Wiley Online Library, clinicaltrials.gov, Sciencedirect, Google Scholar, Web of Science, WHO International Clinical Trials Registry Platform (ICTRP), patentscope, ARIPO and conference abstracts/proceedings. Search Terms included a combination of keywords such as: genomics, GWAS, pharmacogenomics, -genetics, -kinetics and -dynamics, precision medicine, genes, mutations, African populations, Africa, personalised medicine, and medical subject headings including: cardiovascular disease (CVD), metabolic syndrome (MetS), diabetes, obesity, cancer, neoplasms, obesity, infectious disease, malaria, tuberculosis, HIV, HCV, depression, mental disorder, kidney disease, sickle cell disease (SCD) and RD. A total of 520 papers were retrieved. Thereafter, a meticulous two-step filtration process was performed by at least two investigators assigned to each disease section. First, the titles and abstracts were analysed to retain the pertinent manuscripts. Subsequently, the complete texts of the remaining manuscripts were analysed to extract the information of the relevant variants.

Following filtration, only the pharmacogenetics studies found to have been conducted on African populations and diaspora remained. Subsequently, a template was designed to unify the information obtained from the retained studies. Whenever applicable, collected characteristics from each study included: variant ID, affected allele, gene name, key effect, ethnic group, sample size, drug family, variant frequency, allele frequency and p-values. The completed template was the base for the data presented in the current review. These key results from the extracted studies included in this review are summarised in Supplementary File 1.

**Figure 1.**
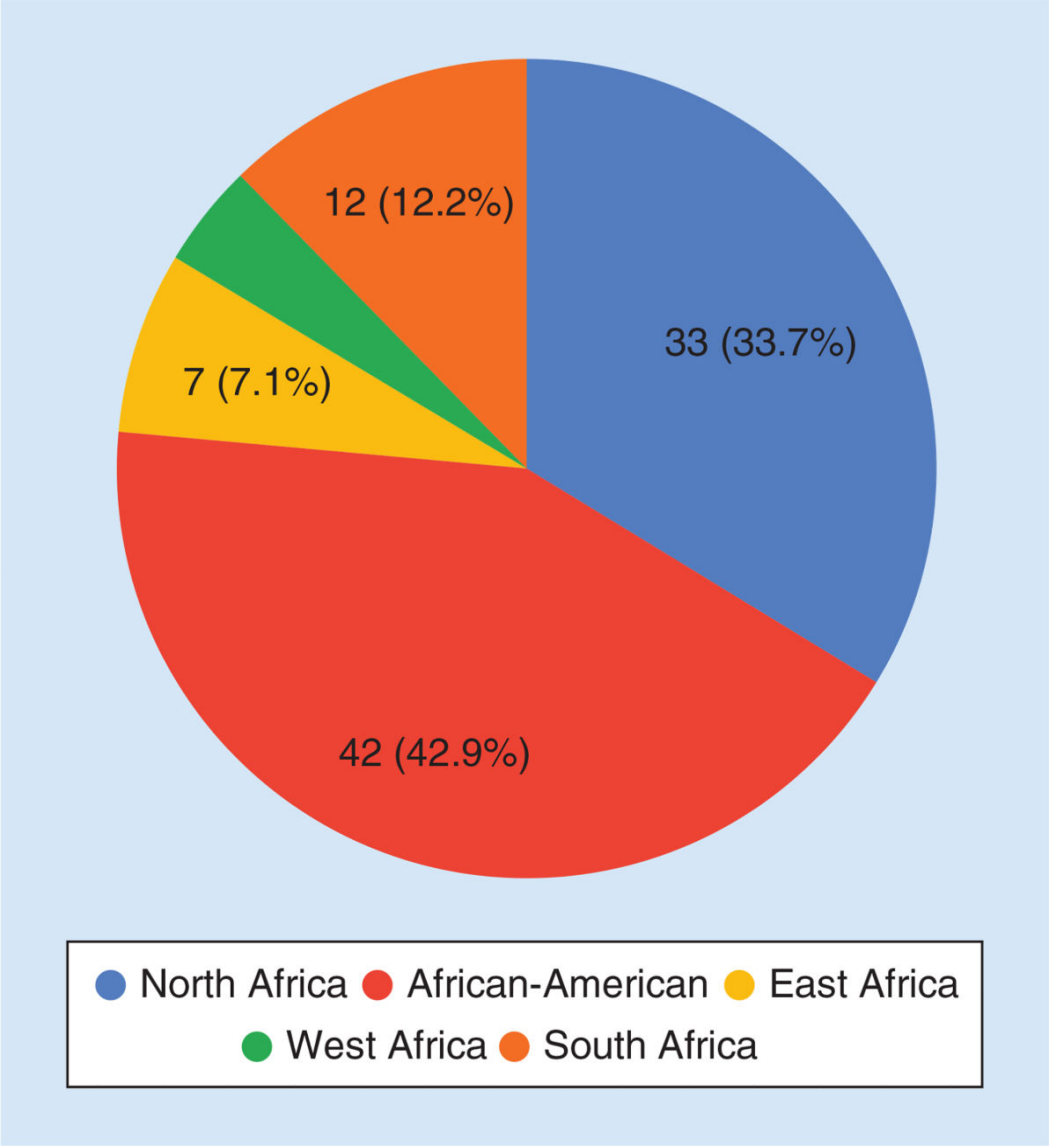
Number of pharmacogenetics studies found to be conducted within various African regions.

**Figure 2.**
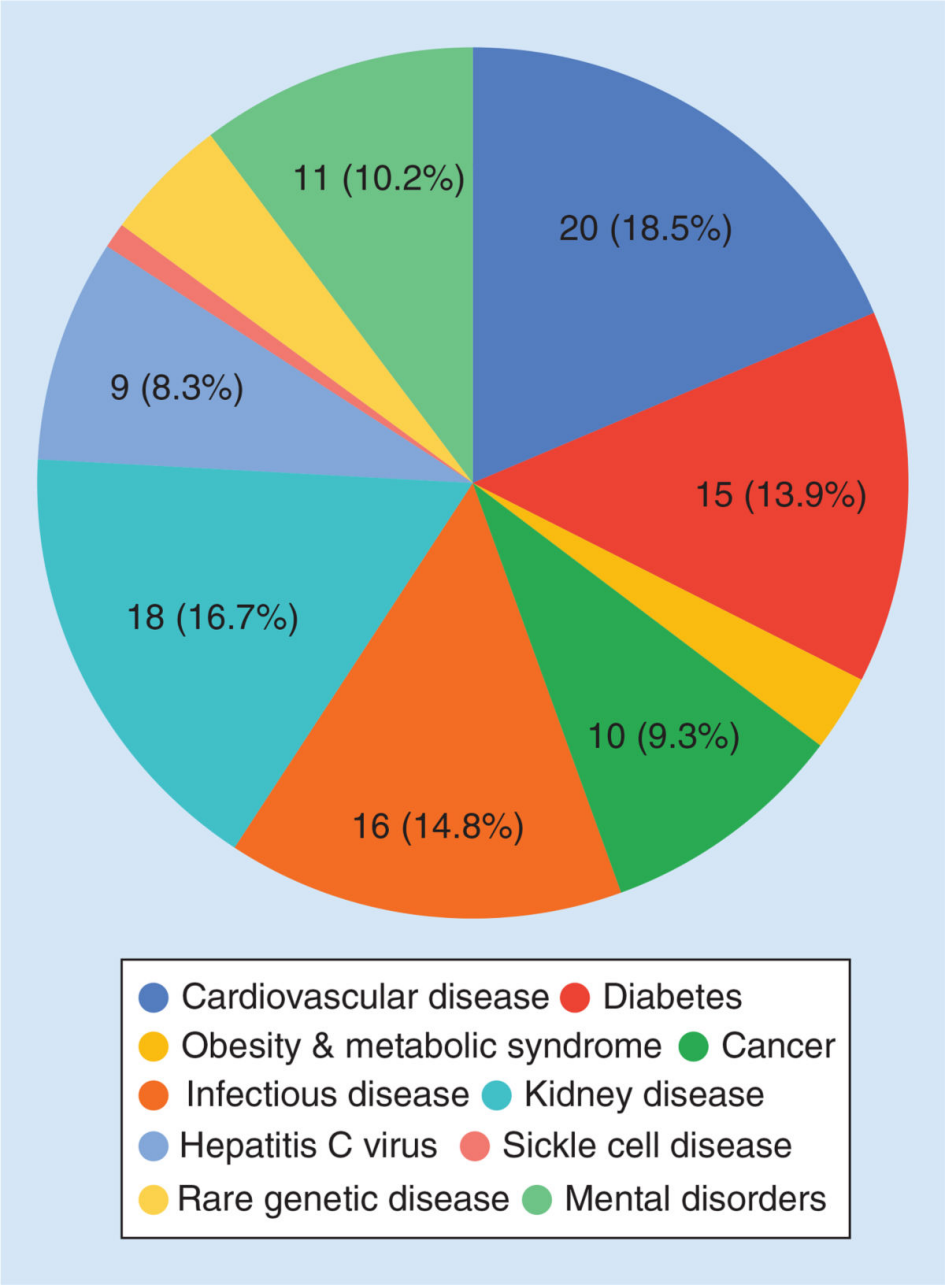
Number of pharmacogenetics studies found to be conducted within various disease fields.

Within Africa specifically, the majority of studies were conducted in North Africa, followed by South, East and West Africa (Figure 1), while the most studied disease fields were CVDs, kidney diseases and infectious diseases (Figure 2). This review does not claim to be comprehensive as new studies are published regularly and some studies may have been missed, but it does represent a large proportion of published studies within the selected geographic regions and disease fields. Hereafter, summaries in each of the disease fields are provided, drugs and countries or populations where or in which the studies were conducted are highlighted in bold in each paragraph.

## CVDs

CVDs are defined as multi-factorial heart conditions and represent the leading cause of morbidity and mortality in both developed and developing countries [6,7]. CVDs are complex disorders, influenced by both genetic and environmental factors [7,8]. Precision medicine is not commonly employed for CVD management; however, 20 CVD-related pharmacogenetics studies were found to have been conducted on African populations.

The majority of these studies focused on warfarin, an anticoagulant that is commonly employed to treat CVD-related conditions. Response to warfarin has been associated with variation in two genes, *CYP2C9* and *VKORC1* [9]. Five studies conducted in Egypt were found. Exploring genetic variation in the genes encoding *VKORC1*, *CYP3C9*, *CYP4F2*, *APOE* and *CALU*, Shahin *et al.* discovered associations between reduced warfarin dose and genetic variants in *VKORC1* (rs9923231), *CYP2C9* (rs1799853, rs1057910) and *APOE* (rs429358, rs7412) [10]. These results were replicated in a separate Egyptian cohort by Bazan *et al.*, who also found associations between rs9923231 (*VKORC1*), rs1799853 and rs1057910 (*CYP2C9*) and reduced warfarin dose [11]. In contrast, rs61742245, another variant in *VKORC1*, has been associated with higher warfarin dose requirements in Egyptian individuals [12]. Interestingly, when examined separately, genetic variants in *ABCB1* (rs1045642), *EPHX1* (rs2234922), and *PZ* (rs2273971) were not associated with warfarin dose, but when combined, these variants were found to influence warfarin dose requirements in the same population [13].

Few CVD pharmacogenetics studies were extracted from the East and South African regions. In Ethiopia, *CYP2C9* haplotypes *1, *2 and *3 (encoded by rs1799853 and rs1057910) had variable frequency, at 94, 4 and 2%, respectively [14]. The effect of several *CYP2C9* haplotypes (*2, *3, *5, *6, *8, *9, and *11), 20 *VKORC1* polymorphisms and clinical covariates were comprehensively assessed in Sudanese patients treated with warfarin [15]. Patients with CYP2C9*2,*5,*6 or *11 haplotypes required reduced daily warfarin dose compared with CYP2C9*1/*1 homozygotes [15]. In contrast to Egyptian studies, no association was observed between rs61742245 in *VKORC1* and warfarin dose in Sudanese individuals [11]. Similarly, rs61742245 was not associated with warfarin dose in a Kenyan cohort either [12]. Interestingly, the variant was not detected in the Ghanian cohort studied [12]. More recently, rs9923231 (*VKORC1*) has been associated with warfarin dose in Ghanians, with GA heterozygotes requiring higher warfarin dose compared with GG homozygotes [16]. In the same study, CYP2C9*2 and *3 haplotypes were not detected [16]. In South Africa, Mitchell *et al.* genotyped *CYP2C9* and *VKORC1* in black South Africans, observing 26 novel and 7 known *CYP2C9* variants, as well as three known *VKORC1* variants [17]. They demonstrated associations between both *CYP2C9* (*8, g.16179 and g.46028) and *VKORC1* variation (rs7200749 and rs7294) and warfarin dosage [17]. These, along with a small subset of environmental factors, explained 45% of warfarin dosage variability in the studied population [17].

The CVD pharmacogenetics has been widely investigated in African-American populations. Genetic variation in *CYP2C9, VKORC1, CYP4F2* and *APOE* has previously been investigated, with observed associations between *CYP2C9* haplotypes (*2, *3, *5, *6 *8, *11) and weekly warfarin dose [18]. The combination of *CYP2C9* variants, *VKORC1* rs9923231 and clinical variables explained 36% of inter-patient variability in warfarin dose requirements in the same study [18]. Additionally, rs339097 in *CALU* has previously been associated with increased warfarin dose in two replication cohorts [19] while novel polymorphisms in *VKORC1* (rs61162043) and *CYP2C9* (rs7089580) have also been associated with increased warfarin dose [19]. Moreover, polymorphisms in *GGCX* (rs10654848) and *CYP2C19* (rs4244285) have been associated with higher warfarin dose requirements [20] and response to clopidogrel therapy [21], respectively. In addition, a genome-wide association study in African-Americans revealed an association between a novel SNP (rs12777823) in the *CYP2C9* and warfarin dose [22].

Clopidogrel is an antiplatelet medication, prescribed to reduce risk of heart disease and stroke. Khalil *et al.* investigated the association between genetic variation in *CYP2C19*, *ABCB1* and *CES1* and clopidogrel response in Egyptian patients with acute coronary syndrome and/or percutaneous coronary intervention [23]. They identified *CYP2C19* variants, age and body mass index as potential predictors associated with variable clopidogrel response [23]. More recently, rs2046934, in *P2Y12*, was associated with clopidogrel response in Moroccan patients with acute coronary syndrome [24]. In Tunisia, Charfi *et al.* investigated the association between the occurrence of adverse cardiac events in patients receiving clopidogrel treatment and CYP2C9*2, however no significant association was observed [25].

Two additional CVD drugs previously investigated are acenocoumarol and rosuvastatin. In Morocco, rs1799853 and rs1057910 (*CYP2C9*), as well as rs9923231 (*VKORC1*), have been associated with weekly acenocoumarol dose, while rs2108622 (*CYP4F2*) was not [26]. Majority of the patients with these variant genotypes were found to belong to a low acenocoumarol dose group [27]. In Tunisia, Ajmi *et al.* demonstrated an association between daily acenocoumarol dose and *CYP2C9* (*2, *3) and *VKORC1* (H1, H7) haplotypes [28].

In Zimbabwe, Soko *et al.* screened 785 individuals from nine ethnic African populations, discovering associations between rosuvastatin exposure and genetic variation in several genes, including the genes encoding SLCO1B1, ABCC2, SLC10A2, ABCB11, AHR, HNF4A, RXRA and FOXA3 [29]. Interestingly, interindividual differences in rosuvastatin pharmacokinetics appeared to be driven by a different set of variants [29].

## Obesity & MetS

MetS is a cluster of multiple metabolic abnormalities that increase risk to CVDs and Type 2 Diabetes (T2D), including obesity, hypertension, dyslipidemia and insulin resistance [30]. The prevalence of these abnormalities in Africa has rapidly increased in the last decade [30]. Three MetS pharmacogenetics studies were extracted from North Africa.

In Egypt, El Sayed *et al.* associated promoter methylation in the genes encoding LEP and MMP2 with folic acid supplementation [31]. Obese children exhibited hypomethylation in this region prior to supplementation compared with post supplementation [32]. In Tunisia, Jmel *et al.* characterised the genetic variability of pharmacogenes previously shown to be involved in MetS drug response [33]. A total of 1056 variants on 24 pharmacogenes were identified in the Tunisian population, while several polymorphisms were associated with anticoagulant sensitivity, including rs3846662 (*HMGGR*) rs1045642 (*ABCB1*), rs7294 (*VKORC1*) and rs12255372 (*TCF7L2*) [34]. Additionally, rs776746 (*CYP3A5*) was also associated with hypolipidemic susceptibility, and rs729 (*VKORC1*) has been associated with warfarin dosage [33,34].

## Diabetes

Diabetes mellitus represents a group of metabolic diseases characterized by abnormal, deficient or inadequate insulin secretion and/or action, resulting in chronic hyperglycemia [35]. Diabetes incidence in sub-Saharan Africa is rapidly rising, ranging from 1 to 15%, however no studies from this region were found [36–38]. Two drugs have been the primary focus of Diabetes pharmacogenetics studies, sulfonylureas and metformin. In the only study extracted from an African country, El-Sisi *et al.* associated genetic variation in *IRS-1* and *KCNJ11* (rs5219) with Sulfonylureas efficacy in an Egyptian cohort [39].

Several studies containing African-American individuals, explored the interaction between candidate genes and metformin as treatment or therapeutic intervention for T2D. The A allele of rs12943590 (*SLC47A2*) has previously been associated with increased renal and secretory clearance of metformin [40] as well as reduced metformin response in African-American T2D patients [41]. Similarly, *SLC47A2* variation (rs2252281 and rs12943590) have also been associated with metformin efficacy and metabolism [40]. In addition, multiple variants in *SP1* (rs784892, rs2683511, rs10747673 and rs784888) have been associated with metformin efficacy and metabolism, while rs149711321 (*PPARA*) has been associated with altered metformin response in T2D patients [42]. Moreover, renal clearance of metformin has been shown to be significantly greater in healthy African-American volunteers heterozygous for rs316019 (*OCT2*) than those homozygous for the reference allele [43]. Several additional candidate genes have been either nominally associated or not associated with metformin intervention in an African-American cohort included in the Diabetes Prevention Program [32,44–50]. These include the genes encoding SLC22A2, HNF1B, ABCC8, ENPP1, TCF7L2, WFS1, ATM, SLC30A8, PPARG, and more.

## Cancer

The molecular landscape of cancer differs by geographical location and genetic ancestry; African-American individuals have been found to have 25% higher cancer mortality rates than Caucasian Americans [51]. A couple of African studies have been performed to link genetic data with response to drugs used for pain in cancer patients. Unlike previous studies conducted on Caucasian and Chinese populations, no significant associations were found between polymorphisms in *OPRM1* (rs17174629, rs1799972 and rs1799971) and *COMT* (rs4680), and opioid treatment for pain in Tunisian cancer patients [52]. A study In Ethiopia revealed that a high proportion of the population are rapid codeine metabolisers due to *CYP2D6* polymorphisms, resulting in rapid conversion of codeine to morphine and subsequent therapeutic overdoses [53].

There are a few African pharmacogenetics studies on chemotherapy drugs, with varying results observed. For example, in Tunisia, resistance to anthracycline-based chemotherapy was found to not be associated with variation in either genes encoding MDM2 (rs1196333) or TP53 (rs1042522) variation [54]. In African-Americans, carriers of *DPYD* variants have been shown to be predisposed to hematologic toxicities when treated with 5-fluorouracil compared with Caucasian-Americans, while Caucasian-Americans are more likely to suffer from diarrhea, nausea, vomiting and mucositis compared with African-Americans [55].

Several African studies have explored the pharmacogenetics of chronic myeloid leukemia (CML) treatment. In Tunisia, Ben Hassine *et al.* reported no significant association between imatinib therapy and *ABCB1* in CML patients, neither at genetic variant nor transcriptional level [56]. In contrast, *ABCB1* (rs1045642) has been associated with lower through plasma concentration of imatinib in Nigerian CML patients [57]. In Egypt, though no association was observed between *SLCO1B3* and imatinib response, a *CYP3A5* haplotype (*3) was associated with treatment failure [58]. In another Egyptian study, rs2032582 (in *ABCB1*) was associated with imatinib sensitivity and resistance [59]. Within the same gene, a haplotype was identified associated with lower probability of achieving optimal therapeutic response [60]. Interestingly, *OCT1* expression has been suggested as a clinical biomarker for imatinib response, as the gene was significantly downregulated in Tunisian samples from the imatinib-resistant group compared with the imatinib-responder group [61]. In additiion, only one African study focused on breast cancer therapy; Abdeljaoued *et al.* showed that male Tunisian breast cancer patients with high *FOXM1* expression exhibited significantly lower response rates to chemo- and hormone therapy than those with low *FOXM1* expression [62].

## Infectious diseases

Africa has a disproportionate burden of infectious disease, with the major killers being malaria, tuberculosis (TB) and HIV/AIDS. As an example, the continent has the highest proportion of individuals exposed to *Plasmodium sp.,* with 81.7% of registered malaria cases and 92.6% of deaths in the world [63]. Yet only one study on malaria treatment study was found. In this study, genotyping was used to distinguish recrudescence from new malaria infection in Uganda. Efficacy, safety and risk of recurrent parasitemia was compared between artemether-lumefantrine treatment and dihydroartemisinin-piperaquine alternative therapy. Alternative therapy was described as highly efficacious and incorporated in the national antimalarial treatment policy [64].

Increased focus has been placed on the pharmacogenetics of TB and HIV. TB affects over 10 million people worldwide [65], with poor outcomes exacerbated by co-infection with HIV. HIV/AIDS remains a serious global health concern, with over 25.6 million cases in sub-Saharan Africa alone [66,67]. Efavirenz is commonly employed as HIV anti-retroviral, however, a genetic variant in *CYP2B6* causes efavirenz to be metabolised at reduced rates. It has been estimated that up to 50% of individuals of African descent infected with HIV have this genetic variant [4]. Currently South Africa has the highest number of patients on antiretroviral therapy (ART) and thus, multiple studies have been conducted on ART’s efficacy. A recent study characterising HIV-infected children for *CYP2B6* polymorphisms, identified a T-G-T haplotype which predicts efavirenz plasma concentration in black South African children [68]. Similarly, polymorphisms in *ABCB1* (rs2032582 and rs1128503) have also been linked to efavirenz concentration [69]. These polymorphisms are also contained in an *ABCB1* haplotype (T-G-T-A) associated with increased plasma efavirenz levels [69]. In Botswana, Gross *et al.* identified associations between *CYP2B6* variants (rs3745274 and rs28399499) and efavirenz-based treatment outcomes among HIV-infected patients [70]. They identified slow metabolism alleles which were associated with reduced clearance but not with the treatment end points [70]. A study in Congo also investigated the distribution of rs3745274 genotypes in patients receiving efavirenz treatment. The CYP2B6*GG (rapid metabolizer) genotype was observed in 17% of Congolese individuals, while GT (intermediate metabolizer) and TT (poor metabolizers) were observed in 55 and 28% of individuals, respectively [71]. Recently the Clinical Pharmacogenetics Implementation Consortium published a comprehensive review on *CYP2B6*, providing dosing guidelines for different age groups based on genotype [72].

While studies in HIV have focused primarily on efavirenz pharmacogenetics [73,74], those in TB have looked mostly at isoniazid and rifampin. Ben Mahmoud *et al.* investigated the association between *NAT2* haplotypes and antituberculosis hepatotoxicity induced by isoniazid in Tunisian patients [75]. They discovered the existence of slow acetylation profiles in southern Tunisia, exhibiting higher incidence of isoniazid-induced hepatotoxicity, while fast acetylation profiles were associated with treatment failure. These results were replicated In another Tunisian cohort, where *NAT2* (*5,*6, *4,*12 and *7) haplotypes and *CYP2E1* (rs2031920, rs3813867 and rs6413432) were associated with isoniazid treatment response [76]. In West Africa, Dompreh *et al.* examined the relationship between genetic variation in *NAT2* and *SLCO1B1,* and isoniazid and rifampicin pharmacokinetics, respectively, in Ghanaian children with TB [77]. They discovered that *NAT2* and *SLCO1B1* genotyping had minimal clinical utility due to *NAT2*’s modest effect and the rarity of the *SLCO1B1* polymorphisms in the population [77]. In east Africa, Weiner *et al.* discovered that rs11045819 in *SLCO1B1* is associated with lower rifampin exposure in TB patients, while Chigutsa *et al.*, reported that the rs4149032 (in the same gene) is common and also associated with low-level rifampicin exposure in TB patients from southern Africa [73,78]. However, these results were not replicated in Malawi [78].

With the frequency of TB-HIV co-infection, there is potential for interaction between TB therapies and ART. This is evidenced by the discovery of a modest decrease in mean efavirenz plasma exposure with rifampin co-administration in healthy African-American and Caucasion volunteers [79]. *NAT2* genotypes have been associated with isoniazid hepatotoxicity, but also with fast, intermediate and slow acetylation of efavirenz in South Africa [79]. In Zimbabwe, a *CYP2B6* haplotype (*18) was associated with reduced efavirenz clearance and elevated plasma concentration [80]. Similarly, the high frequency of this haplotype was associated with decreased metabolism of efavirenz in South African co-infected patients [81].

HCV is another major infectious disease with over 200 million cases worldwide [82]. HCV genotypes are widely distributed by region and ethnicity for example, HCV genotypes 1, 2, 3 and 4 are frequent in North Africa and the Middle-East [83], while HCV genotype 5 is prevalent in southern Africa [80]. Standard treatment for HCV consists of a combination of pegylated interferon (PEG-IFN) α and ribavirin (RBV) [84]. An association between *HLA-A1* and susceptibility to viral clearance (SVR) following PEG-IFN/RBV therapy was previously reported in Egyptian patients [85]. Similarly, *IL28B* (rs12979860 and rs8099917) variants have also been associated with SVR rate following PEG-IFN/RBV therapy [86]. Derbala *et al.* also investigated the impact of *IL28B* polymorphisms (rs12979860, rs8099917 and rs11881222) in response to treatment in Egyptian patients with genotype 4 [87], suggesting them as pre-treatment biomarkers. Fathy *et al.* reported a 46% treatment response value for *IL28B* rs8099917 in predicting SVR among HCV-infected Egyptian patients treated with PEG-IFN/RBV [88]. In sub-Saharan Africa, an association between treatment response and rs12979860 was observed in HCV genotype 4 infected patients [89]. They showed that the treatment response rates among the different ethnic groups (Egyptian, European and sub-Saharan Africa) were 81.8, 46.5 and 29.4%, respectively [89].

In African-American samples, McCarthy *et al.* reported rs12979860 (*IL28B*) as the strongest PEG-IFN SVR pre-treatment predictor in HCV-infected patients [90]. Notably, Thomas *et al.* found that African-American patients displayed lower SVR rates than Caucasian patients despite having the same *IL28B* genotype [91]. Pagliaccetti and Robek further explained that *IL28B* polymorphisms (rs8099917 and rs12979860) are strongly associated with HCV clearance, noting that genotypes associated with poor response to therapy are found at higher frequency in African populations compared with European populations [92].

## Kidney diseases

Kidney diseases are chronic or acute, involving damage to or disease of a kidney [93]. Majority of the pharmacogenetics studies have focused on drugs employed in kidney transplant procedures, such as cyclosporine. In Egypt, the rs4646437 (in *CYP3A4*) has been associated with cyclosporine therapy in individuals receiving kidney transplantation [94]. Similarly, rs2032582 (in *ABCB1*) has been associated with altered cyclosporine dosage requirements [95]. Notably, the variant was not associated with risk of transplant rejection during cyclosporine therapy [95].

Another drug used following kidney transplants is tacrolimus, an immunosuppressant. In Morocco, the rs776746 (in *CYP3A5*) has been associated with altered tacrolimus dose requirements in individuals receiving kidney transplantation compared with those with genotype *CT* [96]. Similarly, *CYP3A5* haplotypes have been associated with decreased trough and dose-adjusted trough concentrations of tacrolimus in Tunisian**s** receiving kidney transplantation [95].

Haplotypes in *CYP3A4* and *CYP3A5* have been extensively explored with regards to tacrolimus therapy in the USA. These include *CYP3A5* haplotypes (*1, *3A, *6 and *7) which have been associated with decreased tacrolimus clearance, increased doses of tacrolimus, increased risk of transplant rejection and delayed graft function, and increased glomerular filtration rate in African-Americans receiving kidney transplantations [97–99]. Notably, these, along with an *ABCB1* variant (rs1045642) were not associated with drug toxicity or concentration [97]. Notably, though several *CYP3A4* and *CYP3A5* variants have been investigated with regards to clinical outcomes of individuals receiving kidney transplants, no associations were observed (rs2740574, rs2246709 and rs776746) [6]. Two *CYP3A4* variants (rs2246709 and rs2740574) have been associated with amlodipine efficacy in African-Americans with hypertensive renal disease [100]. Similarly, an *ADRB2* variant (rs2053044) has been associated with ramipril efficacy in African-Americans with hypertensive renal disease [101].

Several additional studies, investigating tacrolimus therapy in kidney transplantations included African-American samples, however these numbers were either not defined or low compared with more focused investigations [101–104]. Two mixed ethnicity studies with African-American individuals associated rs776746 (in *CYP3A5*) with tacrolimus trough concentrations [101], and tacrolimus dose [102,103] in individuals receiving kidney transplantations. An additional mixed ethnicity study conducted by Pulk *et al.*, associated *CYP3A5* haplotypes with tacrolimus trough concentrations in kidney transplant recipients [104].

## SCD

SCD is the most common recessive single gene disorder in the world, affecting the structure and function of hemoglobin [105]. Hydroxyurea is the only US FDA approved SCD treatment, however response has been associated with genetic variation [105]. Only one pharmacogenomics study was identified in Africa; in Egypt, no associations were found between genes encoding GSTM1, GSTT1 and GSTP1, and hydroxyure**a** treatment [106].

## Rare genetic diseases

Rare diseases (RD) are life-threatening or chronically debilitating heterogeneous diseases which are of such low prevalence that there is often a lack of available knowledge and drugs developed to treat these disorders, and special combined efforts are required to address them [107]. RD of genetic origin constitute a serious health burden in developing countries, and little is known regarding their spectrum in African populations. In Tunisia, improved cochlear implant outcomes have been observed in deaf individuals for whom the aetiology of hearing loss is related to *GJB2*) mutations and who were implanted at an early age [108]. Similarly, primary hyperoxaluria type 1 patients that present with the Maghrebian founder mutation p.I244T have been shown to be pyridoxine non-responsive and therefore, the only therapeutic strategy is combined liver and kidney transplantation [109,110]. Lumacaftor, used for cystic fibrosis (CF) treatment, has also shown potential treatment benefit in African-American CF patients. For an African-American CF patient, with a variant in the gene encoding CFTR, Zhang *et al.* tested response *in vitro* [111]. The variant resulted in a significant increase in total *CFTR* protein expression and channel function [108]. Interestingly, in another African-American CF patient, *CFTR*-expressing cells also responded positively to the *in vitro* addition of lumacaftor [112].

## Mental disorders

Mental disorders are characterised by behavioural or mental patterns that cause significant distress or impairment of personal functioning. In the absence of change in current prevalence rates, estimates suggest that sub-Saharan Africa will experience an increase in the burden of mental and substance use disorders of approximately 130% in the future [113].

### Schizophrenia

Schizophrenia is a severe mental disorder which may lead to delusions, hallucinations and loss of reality, and is generally treated with anti-psychotics. No pharmacogenomics studies in this context were found in North, East and West Africa. The only study found was conducted in South African schizophrenia patients [114]. A frameshift variant, rs11368509, in *UPP2* conferred to improved response to anti-psychotics in mixed ancestry and Xhosa populations [114].

A number of additional schizophrenia pharmacogenetics studies have been conducted in African-American individuals. A study assessing genetic variants in treatment-intolerant schizophrenia patients found that three *DRD2* variants were associated with improved response to clozapine in African-American individuals [115]. Similarly, two variants (rs909706 and rs742105) in *DTNBP1* have also been linked to clozapine and haloperidol response in a mixed cohort of schizophrenia patients [116]. Similar associations were observed between rs165599 (in *COMT*), rs724226 (in *GRM3*) and improved risperidone response in African-Americans compared with European populations [117].

Although individuals of African ancestry have exhibited improved response based on genetic factors, studies have indicated that these populations are at greater risk of antipsychotic-induced weight gain. Genetic variation in *CNR1* (rs1049353) has been associated with higher percent weight gain in African-American patients, compared with European-Americans [118]. Other marginal findings include variants in *PTPRD* [119], *IL1B* [120] and *INSIG2* [121].

### Major depressive disorder

Major depressive disorder is a mental health disorder characterized by persistently depressed mood or loss of interest in activities, causing significant impairment in daily life [122]. No pharmacogenomics studies in this context were extracted from Africa. However, a recent review highlighted eight genes implicated in antidepressant treatment response in mixed populations (including African-Americans). These included *CYP2D6, CYP2C19, SLC6A4, ABCB1* (rs2032583 and rs2235015), *FKBP5* (rs1360780, rs3800373 and rs4713916); *GNB3* (rs5443); *BDNF* (rs6265); and *HTR2A* (rs7997012 and rs6313) [122]. Another study used genome-wide single nucleotide polymorphism data to examine independent contributions of race and genetic ancestry to antidepressant response [123]. Genetic African ancestry predicted lower treatment response in all models [123]. Finally, rs10473984 in *CRHBP* has been associated with both remission and reduction in depressive symptoms in response to citalopram, African-American [124]. This association particularly pronounced in patients with features of anxious depression [124].

## Discussion & future perspective

In recent times, precision medicine and pharmacogenomics have become cornerstones of healthcare in some developed countries, and an important avenue of research to improve the patient’s treatment and management. In this review, relevant pharmacogenetics studies on individuals of African ancestry were retrieved, however, a relative lack of information and data for many African populations were also revealed. Some regions are better studied than others, as are some disease treatments. Though this review does not claim to be comprehensive, several key points have emerged.

First, it is evident that studies on genetic associations with drug response in African populations are less abundant than for other populations, which may be due to limited funding for such studies in the generally more poorly resourced institutions in Africa, as well as overall lack of capacity. Of the >300 medicines with FDA pharmacogenetic product label information [125], and 100 medicines (small molecules) with clinical pharmacogenetics guidelines [126], this review shows that only 15 (warfarin, clopidogrel, acenocoumarol, rosuvastatin, anthracycline, codeine, 5-fluorouracil, imatinib, efavirenz, isoniazid, tacrolimus, clozapine, risperidone, haloperidol and citalopram) have been studied in African populations, and each, in an average of 1–3 studies in the vast continent of 54 countries, over 1 billion people and thousands of ethnically diverse populations. There is a lack of population-specific pharmacogenetics tests and dosing algorithms due to both limited capacity and cohesion of genomics research and clinical pharmacology expertise on the continent. Clinical pharmacogenetics tests are often too costly for low resource settings, and limited laboratory and health informatics infrastructure hamper the ability to offer these tests. The uneven geographic distribution of pharmacogenetics studies also reflects the unbalanced participation of African populations in clinical trials. If more clinical trials were run in low-to-middle-income countries with a high disease burden, there will be an increased likelihood of new therapies being appropriate for implementation in those countries. Additionally, if more clinical trials included a genetic component, our pharmacogenomics knowledge base would increase substantially.

Second, in addition to limited pharmacogenomics studies or clinical trials in Africa, we have a general lack of large-scale genetic studies which can contribute to data on background reference populations. More data are needed on which polymorphisms are truly novel, rare or common in some populations. There have been recent studies on variants in ADME (absorption, distribution, metabolism and excretion) genes in different healthy populations which are helping to identify variants and their frequencies that may be relevant in pharmacogenetics. In a study of sequence data from 40 South Africans of Bantu ancestry, 1662 variants were identified in 65 ADME genes, some of which were novel and a few were potential loss-of-function variants [127]. The novel variants may be important for moderating treatment outcome, but their effect still needs to be determined. The authors also highlight the need for a more comprehensive understanding of population-specific differences to implement pharmacogenetics approaches to treatment.

A third challenge is in identifying relevant studies through clarity on which ethnic group was involved in the study. Racial and ethnic categories are not always consistently reported in different studies [128]. Zhang and Finkelstein, in searching the literature for pharmacogenomics/pharmacogenetics papers, found heterogeneity in classification of ethnic categories with 62 different categories for ‘Black’ [128]. While classifying people into ethnic groups can raise racial issues, in pharmacogenetics, it is essential due to the vast differences in allele frequencies in highly relevant polymorphisms across different populations. Finally, there is still a lack of evidence-based clinical studies that are sufficiently powered (in terms of prevalence of pharmacogenetics variants or effect size of variants on clinical phenotypes) to detect and quantify clinical pharmacogenetics in African populations.

A fourth challenge is the lack of pharmacokinetics studies in people of African ancestry on both old and new medicines. It is important to note that the discovery of pharmacogenetics variability was driven by observations in variation in drug exposure pharmacokinetics studies (debrisoquine, isoniazid, etc) and/or associated with clinical responses (response, failure of response or drug toxicity). African institutions lack strong clinical pharmacology departments with bio-analytical and pharmacometric expertise to conduct such studies. Recent establishment of Phase I clinical trial facilities, some of them with strong bio-analytical capabilities should start addressing this gap [129]. This is because the current sequencing driven initiatives might continue to discover potentially functional genetic variants but with no phenotype (drug exposure and/or clinical effect) data to correlate with. Similar efforts in clinical pharmacology (pharmacokinetics and pharmacodynamics) studies need to be applied as those being put to population genetics/genomics work, only then can a meaningful pharmacogenomics outcome for precision medicine emerge.

A fifth issue that needs to be addressed as we go forward is the need to quantitatively evaluate the unexplained pharmacokinetic/pharmacodynamic variation gap when current European ancestry clinical pharmacogenetics tests are applied to African patients. This is against a current theoretical argument that the unique genetic variation continuously being discovered in African populations will affect clinical outcome significantly compared with outcomes observed in Caucasians. While it makes theoretical sense, given the controversies around the clinical impact/relevance of some pharmacogenetics markers even among the Caucasian populations [130], some observed genetic differences uniquely observed in African populations might easily disappear in the noise of the numerous factors (such as drug/food–drug interactions, disease–drug interactions, drug-biometric indices differences) that can affect clinical pharmacokinetic/pharmacodynamic outcomes. There is, therefore, a need to evaluate, in well powered studies, the currently used Clinical Pharmacogenetics Guidelines for selected drugs and apply them to African patient groups and determine their level of success or failure in predicting and/or reducing risk of adverse effects or treatment failure. Discovery genomics will then be driven by studying patients in whom the current Clinical Pharmacogenetics Guidelines will have failed to predict outcome in the African populations.

At last, but not least, issue to be addressed, is the need to continue applying for, and investing funds to prepare African scientists, healthcare personnel and specialized institutions for facing the challenge of transition from traditional to precision medicine approaches. As progress with regards to pharmacogenetics and precision medicine is taking confident steps all over the world, closely involved teams and facilities in Africa should be both vigilant and confident enough to actively participate in this era. There is a need to improve the knowledge scope and capabilities of both healthcare personnel and facilities concerned with genetics and genomics, population studies, precision medicine and well-designed clinical trials. This need will only be accomplished by integrative initiatives of African and International involved parties. To address these concerns, more synergy between African institutions in terms of African pharmacogenetics and precision medicine research is needed. Data and sample sharing are essential for accelerating scientific progress. Several ethical and socioeconomic challenges need to be resolved such as community engagement, informed consent, possibilities of genetic discrimination and stigmatisation and data security.

Fortunately, some of these challenges are being addressed through funding initiatives for genomics projects in Africa (e.g., H3Africa [131]), and the collaboration between cohorts, public data repositories and standards initiatives such as GA4GH [132], to encourage better curation of metadata associated with genomic and patient data. With the cost of genotyping and sequencing dropping, in 5 years it may be feasible, even in some African countries, to genotype all patients who are prescribed therapies with known pharmacokinetic or genetic variability prior to treatment. This will not only benefit the patient with more precise medication, but also save the healthcare system, the costs of inadequate treatment or the need to treat adverse side effects. In this way, the genetically diverse African populations would benefit from pharmacogenomics-based healthcare approaches to reduce drug side effects and optimize drug choices and doses for each patient. This will require several key components, including the implementation of policies that promote precision public health, such as ethical and legal procedures for the management of communicable and noncommunicable diseases. The policies must clearly articulate support for accurate diagnosis and treatment and should also include pharmacovigilance programs to ensure reporting of new cases of adverse drug reactions. Despite the dropping costs of sequencing, to achieve this in a shorter time frame, cost effective population-relevant panels for screening need to be developed, for which we need more data on pharmacogenetics of African populations. If pharma, research funders and governments are willing to invest in large-scale pharmacogenomic studies, possibly alongside clinical trials, then effective generic or disease-specific pharmacogenetics screening panels will be perfectly feasible in the near future even in resource limited countries.

## Supplementary Material

Supplementary file

## Executive summary

- Pharmacogenetics studies in African populations are less abundant than for other populations.
- Several studies are available on warfarin pharmacogenetics and dosing requirements.
- There is a lack of pharmacogenetics studies for some diseases, such as sickle cell disease.
- Pharmacogenetics studies for infectious diseases have focused on HIV (efavirenz) and TB (rifampin).
- Disease-specific pharmacogenetics screening panels may be feasible in the future.
- More precise medication for African patients can save the healthcare system the costs of inadequate treatment or the need to treat adverse side effects.
